# Geriatric Assessment in Older Patients with Advanced Kidney Disease: A Key to Personalized Care and Shared Decision-Making—A Narrative Review

**DOI:** 10.3390/jcm14051749

**Published:** 2025-03-05

**Authors:** Elisabeth J. R. Litjens, Melanie Dani, Wouter R. Verberne, Nele J. Van Den Noortgate, Hanneke M. H. Joosten, Astrid D. H. Brys

**Affiliations:** 1 Department of Internal Medicine, Division of Nephrology, Maastricht University Medical Centre+, 6229 HX Maastricht, The Netherlands; elisabeth.litjens@mumc.nl; 2 Department of Geriatrics, Hammersmith Hospital, Imperial College Healthcare NHS Trust, London W2 1NY, UK; melanie.dani@nhs.net; 3 Department of Internal Medicine and Geriatrics, University Medical Center Utrecht, 3584 CX Utrecht, The Netherlands; w.r.verberne-3@umcutrecht.nl; 4 Department of Geriatric Medicine, Ghent University Hospital, 9000 Ghent, Belgium; nele.vandennoortgate@ugent.be; 5 Department of Internal Medicine, Division of General Internal Medicine, Section Geriatric Medicine, Maastricht University Medical Center+, 6229 HX Maastricht, The Netherlands; hanneke.joosten@mumc.nl

**Keywords:** end-stage kidney disease, older patients, treatment pathways, geriatric assessment, shared decision-making

## Abstract

As the global population ages, so too does the prevalence of older people with chronic kidney disease (CKD). Helping people age well with CKD and supporting older people with end-stage kidney disease (ESKD) to make personalized decisions regarding kidney replacement therapy (KRT) or conservative care (CC) are an essential component of care. However, these factors are relatively underreported in both the fields of nephrology and geriatric medicine, and prospective, randomized evidence is lacking. This narrative review article, authored by both nephrologists and geriatricians, discusses specific geriatric issues that arise in older people with CKD and why they matter. The available evidence for KRT or CC in older people with frailty is outlined. The importance of performing a comprehensive geriatric assessment, or a modified nephrogeriatric assessment, to ensure a systematic evaluation of the person’s medical problems and life needs, goals, and values is described. We consider different models of nephrogeriatric care and how they may be implemented. Kidney supportive care—addressing an individual’s symptoms and overall well-being alongside the more traditional nephrological principles of preventing disease progression and optimizing risk—is highlighted throughout the article. We outline ways of identifying the later stages of a person’s disease journey, when transition to palliative care is indicated, and elaborate methods of preparing patients for this through multidisciplinary advance care planning. Finally, we discuss practice and systems for nephrogeriatric care in five different European countries and consider future directions, challenges, and highlights in this rapidly evolving, increasingly relevant field.

## 1. Introduction

Chronic kidney disease (CKD) refers to a gradual and irreversible loss of kidney function. CKD arises from heterogeneous disease pathways, but diabetes and hypertension are the main causes of CKD globally [1]. The prevalence of CKD increases with age and is estimated to currently affect 9.8% of the global population [2]. As the global population continues to age, the prevalence of CKD has risen considerably over the last few decades, particularly among older adults [3]. Available prevalence data primarily come from annual reports by the United States Renal Data System (USRDS) and the European Renal Association (ERA). In the USA, nearly 33% of individuals aged ≥65 years are affected by CKD [4].

When end-stage kidney disease (ESKD)—defined as an estimated glomerular filtration rate (eGFR) < 15 mL/min/1.73 m^2^) [5]—is reached, kidney replacement therapy (KRT) (i.e., hemodialysis (HD), peritoneal dialysis (PD), or kidney transplantation (KT)) might be required to sustain life over the long term [6]. Advances in healthcare systems and economic progress have made KRT more accessible to a broader range of patients. According to the ERA registry annual report, 20% of individuals receiving KRT are aged ≥ 75 years [7]. Of these patients, the majority undergo dialysis treatment (74% HD and 6% PD, respectively), while 19% undergo KT [7].

Older patients with CKD often face a variety of complex health challenges, including frailty. They typically have multiple comorbidities and may experience functional and cognitive impairments. Additionally, the symptom and treatment burden associated with KRT is substantial. Many patients on dialysis report a health-related quality of life (HRQOL) comparable to that of patients receiving palliative care for cancer [8]. Common symptoms include fatigue, pain, and depressive feelings [8,9]. The social consequences of dialysis treatment, such as frequent hospital visits, social isolation, and increased dependency, might further complicate the management of older patients on dialysis [10,11]. Furthermore, older patients may not be eligible for KT due to comorbidities, limited donor organs, and surgical risks. Dialysis treatment and KT offer the potential to extend life but often at the expense of a diminished HRQOL [12].

In this context, the question arises whether or not older patients with ESKD would benefit more from KRT or whether conservative care (CC) could be a viable alternative. CC offers a treatment pathway that aims to preserve HRQOL with adequate symptom control through active medical treatment and multidisciplinary care [13]. Importantly, CC and palliative care in ESKD are not synonymous. Palliative care, or supportive care, is needed in all patients undergoing KRT, those on CC, and those who choose to discontinue KRT [13,14,15].

To make well-informed decisions, patients and clinicians need accurate, reliable data on patient-relevant outcomes, including survival, disease burden (e.g., hospitalization) and HRQOL domains (e.g., fatigue, pain, physical function, depression, and daily activity), for both KRT and CC treatment pathways [16]. This is crucial as patients often prioritize these outcomes beyond survival when deciding between KRT and CC, thus enabling treatment-decision-making that best aligns with the patient’s values and preferences [17].

However, comparative data on outcomes in KRT and CC pathways are often limited or inconsistent, hindering decision-making. Of these data, survival has been mainly studied, but, so far, only in observational studies. Some studies report that dialysis treatment offers a survival advantage in older adults, particularly for patients with fewer comorbidities or better functional status, even those aged ≥80 years [18,19]. However, others have observed limited or no survival benefit in older patients, particularly not in those with significant comorbidities or poor functional status [20,21,22,23,24,25]. In a recent meta-analysis of 22 observational cohort studies including the above-mentioned studies and covering 21,344 patients, Voorend et al. reported an overall lower mortality risk for patients opting for dialysis compared with patients choosing CC [26].

Recent observational studies examining HRQOL and symptoms in older patients with ESKD choosing either KRT or CC present more consistent findings, suggesting that KRT may not always lead to improvements in HRQOL for older patients [27]. Interestingly, effective CC may preserve or even enhance HRQOL in older patients with ESKD [28]. For instance, Brown et al. showed that although older patients with ESKD opting for CC presented with worse baseline symptoms compared to patients choosing to initiate dialysis treatment, HRQOL improved in both groups after 12 months [24]. Moreover, after dialysis initiation, patients reported lower life satisfaction. So et al. also observed that, although patients receiving CC were older and had more comorbidities and functional impairments at baseline, no significant decline in HRQOL was observed after 12 months, with the HRQOL of patients in the KRT group not changing significantly either [29]. In contrast, Shah et al. noted that older patients undergoing dialysis reported a significantly greater symptom burden compared to those managed conservatively [12]. Additionally, Tamura et al. highlighted that nursing home patients with ESKD initiating dialysis treatment experienced a rapid decline in functional status, which continued for at least one year [30]. Importantly, more than half of these nursing home patients did not survive beyond one year following dialysis initiation. Verberne et al. further demonstrated that patients on CC experienced significantly more hospital-free days annually than those on KRT, contributing to a lower overall disease burden in the CC cohort [31].

The heterogeneity of the available studies, including variations in inclusion criteria such as age, creatinine clearance, and treatment modalities, along with the high risk of biases such as selection bias and lead-time bias [32], makes it difficult to draw definitive conclusions regarding the benefits of KRT versus CC in older patients with ESKD [33]. In most studies, for example, patients choosing KRT tend to be younger and more fit than patients choosing CC, indicating the risk of selection bias and confounding. Whilst this makes the observed similarities in outcomes between both patient groups even more remarkable, such group differences hinder appropriate comparison of whether the outcome results could be explained by the chosen treatment pathway.

Moreover, the available studies often overlook key factors, such as frailty, nutritional status, cognitive function, and psychosocial aspects, which significantly influence disease progression and treatment outcomes in older patients [34,35,36]. Therefore, decisions on initiating KRT or CC in older patients with ESKD should also take these factors into account.

A comprehensive geriatric assessment (CGA) can provide crucial insights into whether KRT or CC is more appropriate for each older patient with ESKD. As a core element of geriatric medicine, CGA involves a multidimensional evaluation of the older patient’s physical, functional, cognitive, and social capabilities, identifies frailty and resilience, and develops an integrated treatment plan tailored to these needs, with ongoing adjustments based on the progression of impairments [37]. A CGA might also help to improve shared decision-making (SDM) and enable person-centered care by identifying and prioritizing the outcomes and goals of care that matter to an individual patient.

**Figure 1:**
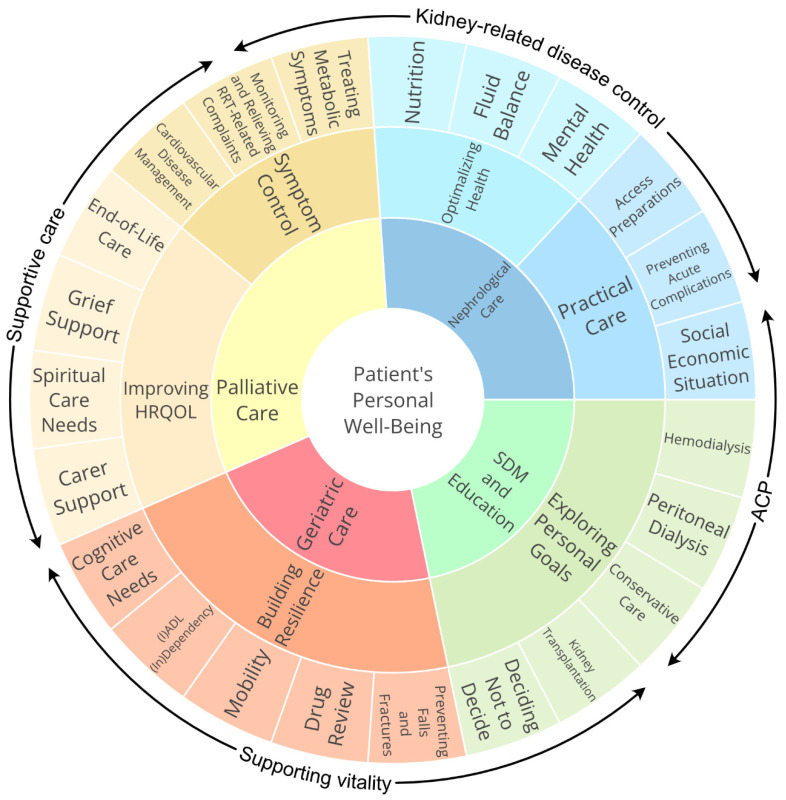
Multidisciplinary approach in the care of older patients with ESKD. ACP: advance care planning; HRQOL: health-related quality of life; SDM: shared decision-making; RRT: renal replacement therapy.

This narrative review discusses the complexity and challenges of managing older adults with ESKD, with a focus on the decision-making process between KRT and CC. It will incorporate insights from both nephrologists and geriatricians, underscoring the importance of collaborative care in these patients. Figure 1 summarizes the various aspects relevant to the care of older patients with ESKD. The review will also discuss tools, such as the CGA and modified ‘renal-CGAs’ (also called nephrogeriatric assessment (NGA)), that help to address critical issues such as frailty, cognitive decline, functional impairment, psychosocial challenges, and resilience, all of which influence disease progression, treatment outcomes, and SDM in this patient population. Furthermore, we demonstrate how to identify older patients with ESKD who may benefit from transitioning to palliative care. Lastly, this review will provide an overview of current CKD care strategies and guidelines across five European countries, including current collaborative nephrogeriatric care practices.

## 2. Background

‘Frailty’ is defined by the Oxford English dictionary as ‘the quality of being physically weak or fragile, or vulnerable to damage; the state of being mortal’ [38]. The biological syndrome of frailty refers to reduced physiological reserves arising from dysregulation across multiple body systems. These reduced reserves lead to increased vulnerability and impaired resilience to stress [39]. Frailty is separate from, but often associated with, multimorbidity and ageing. It is a dynamic marker that can track interventions such as medical treatment, exercise and nutrition [40,41,42]. It is associated with a range of ‘geriatric syndromes’—conditions which do not fit defined disease categories, but which represent a higher order complex system failure and are often a manifestation of a wide range of underlying conditions [43]. They include falls, delirium, pressure ulcers and incontinence [44].

Frailty is common in older patients with CKD. One meta-analysis found that 73.5% of patients with CKD have some degree of frailty or pre-frailty, compared with 63% of the general population [45]. It is present in 40% of patients on HD [45] and in 40% of patients with ESKD [46]. As in other conditions, frailty in CKD confers worse HRQOL and increased risk of falls, fractures, and depression [47,48]. In patients on HD, it is associated with more hospitalizations and emergency events. It is consistently associated with increased mortality in patients across different stages of CKD [35,49,50]. Older adults with CKD are more likely to develop frailty than older adults without the disease [51]. Additionally, the rate of eGFR decline correlates with incident frailty, suggesting that aspects of the disease process intrinsically accelerate frailty progression, such as inflammation, metabolic acidosis, anemia, vitamin D deficiency, and an overall catabolic state [52].

The physical trajectories of individuals with frailty can be seen as a slow, meandering deterioration from a low baseline, in contrast to more precipitous declines in those with more defined diseases such as cancer [53]. As such, individuals with frailty, as well as those with ESKD, who are coming to the end of their lives can be difficult to recognize. One large cross-sectional study showed that patients with frailty, ESKD, or cardiopulmonary failure are less likely to have access to palliative care at the end of life, less likely to have family reports of good end-of-life care, and are more likely to die in an Intensive Care Unit than those with dementia or cancer [54].

## 3. A Balanced Perspective: The Nephrologist’s Versus the Geriatrician’s Point of View

### 3.1. The Nephrologist’s Perspective

Guidelines regarding CKD management mainly focus on the correction of metabolic complications, preventing progression to ESKD, preparing patients for KRT, and management of cardiovascular disease. Choosing the appropriate time to start dialysis seems mostly dependent on eGFR and less focused on patients’ goals in life. The process of SDM can help nephrologists meet patients’ needs better and could give patients more control over their CKD management [55]. Patients expect their treating nephrologist to discuss the expected course of CKD in order to make well-informed decisions about starting KRT or choosing CC. Their decision whether or not to start KRT may change in light of a poor prognosis. Notably, several studies have observed a high degree of regret in older patients started on dialysis [56,57]. Hence, openly discussing the prognosis as well as exploring patients’ values and preferences in a SDM process is especially important in older patients.

In recent years, models of patient-centered multidisciplinary care team (MDT) have been proposed. Treatment should be focused on adequate psychological and social support and maximum reduction in symptoms in addition to dietary counselling and medication management. In older patients, special considerations include frailty, multimorbidity, cognitive function, and end-of-life care. The European Renal Best Practice (ERBP) Guideline gives a practical overview of tools and management plans for nephrologists treating older patients with CKD [58]. Optimal care, goals, and treatment targets may change during progression of CKD. Overly strict blood pressure control, for example, may lead to complications. From patients’ perspectives, treatment goals can be more focused on HRQOL rather than prolonging life [59].

With ageing and progression of CKD, cognitive impairment increases, whilst HRQOL and functional status decrease. This is even more prominent after starting dialysis [30,58]. It is therefore important to monitor cognitive and functional status on a regular basis, especially before and after the initiation of dialysis. Programs to help patients to improve and maintain physical fitness are important.

Cognitive impairment is also an important factor for nephrologists to consider when discussing treatment options. During KRT counseling, a considerable amount of information is discussed. Patients with cognitive impairment may experience difficulty understanding or remembering the information given. In such cases, it is necessary to adjust patient education and repeat instructions. Cognitive impairment also has an effect on the dialysis modality choice. Progressive cognitive impairment may, for example, lead to a switch from PD to HD or family members taking over specific executive tasks in PD treatment.

From a nephrologist’s point of view: how do we go about giving older patients with renal failure the best suited care? It is vital to assess the risk of progression to ESKD, the risk of death in general within 6–12 months, and the level of frailty and resilience. The principles of palliative care should be applied for older patients with CKD, using the four dimensions of palliative care model.

Integrating standard renal care with person-centered care that focuses on the specific needs and goals of older CKD patients is challenging and requires coordination and collaboration between different caregivers (Figure 1). Barriers that may rise during implementation are the heterogeneity and multimorbidity of older CKD patients, conflicting individual priorities versus evidence-based outcomes, uncertain benefits and harm, time constraints, and organizational constraints [60].

Therefore, it is important for nephrologists to seek connections and work together with geriatricians in order to learn from their experience and knowledge of the complexity of care for older CKD/ESKD patients. Geriatricians should be an integral part of care teams for these patients and need to be involved in developing a comprehensive care plan. Nephrologists need to consult geriatricians when cognitive function declines and complex treatment decisions have to be made [61].

### 3.2. The Geriatrician’s Perspective

Given the prevalence of frailty in older people with CKD and the consistently higher risk of complications and poor outcomes, a robust skillset in recognizing and managing frailty is essential. Equally important is that the clinician possesses the skills to communicate this to the patient and other healthcare teams. Geriatricians are ideally placed to do this and can support nephrologists by performing a multidisciplinary, individualized assessment (CGA) and setting tailored goals. This has been consistently shown to increase an individual’s chances of being alive and in their own home at follow-up, across a range of hospital settings [37,62].

In renal settings, the geriatrician can support treatment and symptom control for those on non-dialysis pathways and can also focus on associated non-renal frailty conditions, such as cerebrovascular and neurodegenerative disease, falls, orthostatic hypotension, polypharmacy side effects, and challenging glycemic control [63].

Equally important is the comprehensive assessment of comorbidities, particularly cardiovascular diseases such as hypertension and heart failure. Given the strong interplay between kidney function and cardiovascular health, this assessment is essential for guiding both the management and prognosis of these patients. Cardiovascular diseases are prevalent in older adults with CKD and often coexist, creating a bidirectional relationship between the two. Shared risk factors, including ageing, hypertension, diabetes, and dyslipidemia, increase the likelihood of developing both conditions simultaneously. As a result, these comorbidities contribute to poorer outcomes, such as higher mortality rates, more frequent hospitalizations, and diminished HRQOL.

Moreover, a thorough evaluation of pharmacological and dietary treatments is crucial and should be individualized to meet the specific needs of older adults with CKD. Pharmacological interventions must be carefully selected to minimize the risk of polypharmacy-related complications, while dietary strategies should aim to optimize kidney function and prevent malnutrition.

Finally, clear communication with community services, general practitioners, and rehabilitation services and robust knowledge of local resources for social prescribing, physical activity, and cognitive and social networking groups are also vital components of geriatric care. The geriatrician also has an important role in collaborating with nephrologists in guiding SDM and supporting any non-renal conditions appropriately. Additionally, given the consistently poor outcomes in older people with frailty and CKD, geriatricians can play a key role in ensuring that all patients have the opportunity to engage in advance care plan (ACP) discussions. Thus, a collaborative, multi-specialty ‘division of labor’ encompassing differing skillsets can enhance overall care for the patient (Figure 1).

Finally, embedding clinical mindsets and practice into the recognition, management, and communication of the needs of older, frail patients is essential for sustainable, high-quality change. Geriatricians should play a key role in educating trainee clinicians and allied healthcare professionals, sharing findings with nephrologists, and working with the MDT to advocate for the needs of these patients.

## 4. (Nephro) Geriatric Assessment

Nephrology, palliative care, and geriatric medicine are all very familiar with patient-centered care. However, the focus of renal services often centers on symptom control, preventing further renal impairment, managing cardiovascular risk factors, and dietary interventions. While most nephrologists acknowledge the importance of geriatric conditions in patients with advanced CKD, many report a lack of knowledge and limited experience with using geriatric tools [64,65,66].

In relatively recent years, awareness has grown that a more comprehensive and systematic geriatric approach is needed to assess and manage older patients with ESKD. Single frailty screening tools may lack the discriminative power necessary to detect relevant impairments and dichotomous frailty classification has limited utility in clinical decision-making, particularly in identifying patients who need further assessment. On the other hand, a full CGA is time consuming and labor intensive, requiring additional time and personnel on top of standard CKD care [67]. This highlights the need for cost-effective models that integrate both geriatric and nephrological care. Such models would require adapting the CGA to make it more relevant, efficient and tailored to the specific needs of ESKD patients [68]. The added value of geriatric assessment in older adults with CKD is increasingly recognized, leading to the development of modified ‘renal-CGAs’.

Between 2016 and 2019, the first data on structured nephrogeriatric assessment (NGA) emerged from the USA, the UK, and the Netherlands [64,69,70]. In the USA, a CGA-4-CKD program was introduced, which included a six-domain geriatric screening process for older patients with CKD [69]. The Dutch GOLD study evaluated geriatric assessment (GA) as a comprehensive tool for overall health assessment, screening seven domains in older ESKD patients initiating dialysis [70]. In the UK, a Modified Geriatric Assessment (MGA) was developed for dialysis patients, assessing mobility aids, falls, vision or hearing problems, social support, frailty, and cognitive status [64]. These three studies were the first to demonstrate that a GA helps raise awareness and detects geriatric impairments in older CKD patients, leading to the development of the nephrogeriatric liaison specialty [64,69,70].

Since then, nephrogeriatrics has been more widely studied, and the need for multifactorial geriatric assessment is increasingly recognized by nephrologists. Several guidelines now recommend assessing functional and cognitive status, conducting medication reviews, addressing psychosocial aspects, and evaluating fall risk in older CKD patients [58,59,71]. GA is now being studied in various stages of CKD, including ESKD [70], pre-KT [65], dialysis initiation [35], and PD [64]. These studies have demonstrated that GA more clearly identifies the burden of geriatric impairments in older patients with advanced CKD, thereby revealing unmet care and support needs.

### 4.1. Implementation in Health Care

Several studies have explored different nephrogeriatric liaison models and how care pathways can be implemented, depending on local health care systems, expertise, preferences, and resources. Various care models integrating geriatric assessment in renal clinics have been proposed, such as nurse-led or geriatrician-led GAs performed alongside nephrology visits or a GA- or age-based triage for referral to geriatric services for a full CGA [65,69,72,73]. These studies have shown that incorporating NGAs into routine care is feasible.

However, the sustainable implementation of these practices remains challenging [68]. Firstly, implementation requires adequate resources, particularly personnel [68]. Second, nephrologists’ competence and familiarity with geriatric tools are seen as barriers. The lack of geriatric services in specialty clinics hosting dialysis centers is also a concern [66]. Other challenges include patient illiteracy, language barriers, and concerns about patient burden [68]. Facilitators for successful implementation include strong collaboration with geriatric departments, staff educational interventions, multidisciplinary meetings, and assessments conducted by nurses [66,68]. These barriers and facilitators are not unique to renal care, as similar challenges have been identified in other medical fields where GA implementation has been studied, including oncology, pulmonology, surgery, and neurology [74,75,76,77].

### 4.2. The Value of Geriatric Assessment

GA enables the structured identification of frailty and resilience across multiple domains, helping to anticipate potential risks. Frailty is an independent risk factor for both mortality and progression to dialysis [78]. Beyond its prognostic value, GA can guide targeted interventions, such as deprescribing medications, referral to an occupational therapist, or expanding home care services. Since some conditions contributing to frailty are reversible, early identification and intervention can help prevent further decline. For instance, if cognitive dysfunction is detected through GA, it may prompt customized ESKD counselling or trigger additional assistance for medication adherence or fluid restriction management. The primary outcomes of these interventions are improved HRQOL and health outcomes.

Thus, frailty assessment can enhance nephrology practice by facilitating SDM regarding treatment options. Additionally, GA can be used to explore patient priorities and concerns, fostering person-centered care. A GA may also prompt a more comprehensive geriatric assessment (CGA) by a geriatrician, uncovering modifiable issues that go beyond the nephrology evaluation. These insights can be crucial in guiding SDM around treatment decisions and escalation plans.

### 4.3. Future Research Directions for GA

It is crucial to further investigate the efficacy and effectiveness of GA in nephrology practice. Specifically, the impact of GA on treatment decisions, management plan adjustments, and prognostic value for patients with ESKD needs to be evaluated. The ongoing Australian RCT, the GOAL study, is currently assessing the efficacy and safety of a CGA in frail patients with CKD stages 3–5 [79]. Similarly, the Dutch DIALOGICA study is exploring HRQOL and clinical outcomes in older patients, comparing CC versus dialysis, using an NGA to monitor outcomes in geriatric domains [73].

Second, there is currently no gold standard for an NGA. Several studies have proposed different test sets, each combining various screening instruments that assess geriatric domains, often with some overlap in the areas they cover [64,69,70,80]. Interestingly, some NGA test sets also include components such as patient experience, caregiver burden, patient-reported outcome measures (PROMs), medication reviews, and the surprise question. Moving forward, the development of a standardized NGA that integrates the most relevant and effective screening tools could help streamline assessments and enhance consistency in care.

## 5. Transition to Palliative Care

Palliative care is defined by the World Health Organization as follows [81]:

“*The active, total care of patients whose disease is not responsive to curative treatment. Control of pain, of other symptoms, and of psychological, social and spiritual problems is paramount. The goal of palliative care is achievement of the best quality of life for patients and their families*”. Cecily Saunders, the pioneer of palliative care in the UK, stated: “Adding life to years” is far more important than “adding years to life”. Palliative care extends beyond end-of life care, offering a supportive pathway aimed at improving HRQOL for patients with life-limiting illnesses like ESKD, and it can be provided alongside therapies that prolong life, such as dialysis [15].

### 5.1. Palliative Care in Nephrology and Dialysis

A conceptual framework for palliative care in nephrology has been proposed in the USA, which includes two delivery models: primary and specialist palliative care [82]. Nephrologists, nurses, and social workers receive training in palliative care and communication in order to develop embedded kidney palliative care clinics. In Canada, successful integration of a palliative approach into CKD care has been described, focusing on creating a renal network, involving local and regional stakeholders, and fostering a cultural shift through education [83].

**Figure 2:**
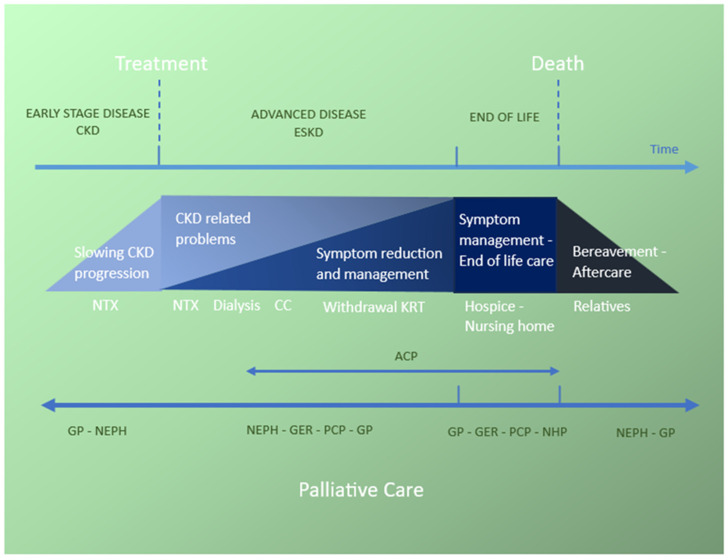
Spectrum of palliative care in older patients along their CKD trajectory. CKD: chronic kidney disease; ESKD: end-stage kidney disease; NTX: kidney transplantation; CC: conservative care; KRT: kidney replacement therapy; ACP: advance care plan; GP: general practitioner; NEPH: nephrologist; GER: geriatrician; PCP: palliative care physician; NHP: nursing home physician. Adapted from original conceptualization by World Health Organization (WHO).

Palliative care in nephrology must be an integral component of care for CKD patients, alongside regular medical interventions, and includes symptom management as well as improving HRQOL, providing emotional, spiritual, and family support, and has to be in line with patients’ values and wishes. Palliative care can be introduced from advanced CKD through to end-of-life care. For patients on dialysis, the approach may evolve from patient-centered dialysis to palliative dialysis, which focuses more on symptom control and HRQOL rather than survival or long-term outcomes (Figure 2). It is important to note that reducing dialysis dose is not synonymous with palliative dialysis, as doing so without careful management may exacerbate symptoms and suffering. Furthermore, palliative dialysis does not automatically equate to the withdrawal of dialysis. The goal of adjusting dialysis prescriptions within the palliative care context is to optimize the patient’s well-being, ensuring that any changes made contribute to symptom relief and improved HRQOL [84].

Not all patients with ESKD benefit from dialysis treatment and may choose a CC pathway instead [27]. The most recent KDIGO guidelines introduce CC as a holistic, person-centered care plan designed to enhance HRQOL for patients with ESKD who opt not to pursue KRT [59]. ACP is a key component of this process.

### 5.2. Advance Care Planning (ACP)

ACP is defined as the process by which patients, in conjunction with their physician and loved ones, discuss and establish wishes, goals, and preferences for future care. ACP focuses on HRQOL and can clarify the preferences for end-of-life care, including ethical, psychosocial, and spiritual issues. ACP in nephrology needs to address wishes regarding dialysis treatment, including when to start, when to stop, and any limitations to interventions such as resuscitation, hospitalization, antibiotic use, and preferred hospice care.

Studies show that older patients prefer to be involved in decision-making regarding end-of-life care [85]. However, physicians often misinterpret patients’ wishes, with poor agreement in up to 80% of cases, especially in scenarios involving treatment preferences [86]. The only way to ensure alignment with (older) patients’ wishes is by directly asking them. More efforts should be directed toward engaging physicians and patients and their families in high-quality discussions when real (not hypothetical) medical decisions must be made. Studies show that older patients’ satisfaction with end-of-life care is strongly associated with their involvement in medical decision-making and the quality of information provided [87].

### 5.3. Initiating ACP Conversations

ACP discussions should be initiated by the healthcare team. Tools that can be useful include the surprise question: ‘Would you be surprised if this patient were to die in the next 12 months’, as well as prognostic tools designed for older populations, such as the Multidimensional Prognostic Index [88] and the e-prognosis website [89]. These tools use individual patient characteristics such as high number of comorbidities (Charlson Comorbidity Index >6), hospitalizations within the prior six months, high level of frailty, hypoalbuminemia, poor nutritional status, and moderate cognitive impairment to guide decisions [90]. ACP discussions should also be offered to patients who choose not to initiate KRT or decide to withdraw from dialysis [91]. When discussing this, it is important to confirm patients’ understanding of their illness and prognosis, ensuring clarity on the outlook for renal failure [91,92].

### 5.4. Challenges in Discussing Prognosis

Discussing illness and prognosis with patients can be challenging, and timing of these conversations might be difficult. The Serious Illness Conversation Guide is a useful tool for structuring these conversations [93]. It offers a six-step framework to help clinicians navigate these discussions and provides examples of patient-tested language to facilitate clear and compassionate communication.

Since CKD is a progressive disease, functional and cognitive decline may occur over time. As patients’ needs, goals, and preferences evolve, it is crucial to regularly reassess and update the care plan in order to make sure that their needs are consistently addressed [84,94]. Palliative care should be delivered by a MDT that includes nephrologists, renal nurses, geriatricians, palliative care experts, dieticians and social workers. Providing effective palliative care in CKD can be challenging, and teaming up with trained palliative care experts helps provide the necessary, multidisciplinary care in CKD and ESKD patients. Depending on the patient’s phase of life and place of care, maintaining communication with general practitioners is also important.

### 5.5. Symptom Assessment and Management

As HRQOL is one of the main goals of palliative care, symptom assessment and management are mandatory. Regular symptom assessment should be conducted using validated tools such as the Edmonton Symptom Assessment System—Revised (ESAS-r), the Renal Symptom Assessment Scale, and the POS-Renal tool. Management should follow a stepwise approach, with available guidelines to support the treatment of uremic pruritus, sleep disturbances, restless legs syndrome, pain, and depression in CKD [59].

## 6. Current CKD Care Practice in Five European Countries—The Highlights

Clinical practices in CKD care vary significantly across countries. In this review, we examined practices in five European countries: the Netherlands, Italy, the UK, France, and Belgium. Table 1 provides an overview of available national guidelines, patient resources, and decision support tools, as well as the composition of MDTs and whether a CGA or NGA is part of standard care per country.

Our key findings highlight that national guidelines for CKD treatment and CC are available in the Netherlands, the UK, and France, while Italy and Belgium primarily follow international guidelines, such as those of the KDIGO, NICE, and ERBP. Dedicated palliative care guidance for patients with ESKD, however, remains less common. Among the reviewed countries, the Netherlands has established a specific guideline, and Italy offers criteria to identify patients requiring palliative care. Expanding such guidelines across countries presents an important opportunity to enhance care for this patient population. Despite the increasing recognition of the need to address frailty, geriatric syndromes, and psychosocial challenges in older patients, none of these countries routinely include a geriatrician in their MDT or integrate a structured GA into standard care for older patients with CKD or ESKD. All countries reviewed provide websites to patients with information about their disease and treatment options. The Supplementary Materials provide a more detailed description of the available guidelines and current CKD practices across the five European countries. Strengthening collaboration among nephrologists, geriatricians, and palliative care experts, alongside harmonizing guidelines, could enhance care quality for patients with CKD across Europe and promote a more comprehensive, personalized approach to managing this patient population.

## 7. Discussion

This narrative review addresses the challenges and complexities in current CKD care for older patients, with a particular focus on decision-making regarding KRT or CC treatment pathways and the importance of person-centered care. We highlight the added value of integrating a geriatric assessment and promoting collaboration among nephrologists, geriatricians, and palliative care experts (Figure 1). Together, these approaches support SDM by incorporating medical, psychosocial, and personal factors into care plans tailored to what truly matters to the older patient with CKD.

### 7.1. Future Directions

In current nephrology care, choosing a CC pathway is increasingly recognized as a viable alternative to KRT for selected older patients, with these treatment pathways becoming more widely available. To improve SDM regarding preferred treatment options, it is essential to gain a better understanding of which older patients are more likely to benefit from KRT versus CC. For instance, cardiovascular comorbidities appear to be strongly associated with a diminished survival benefit in older patients who choose dialysis over CC compared to other comorbid conditions [21,25].

Additionally, and crucially, there is a need for data on patient-relevant outcomes beyond survival alone, such as outcomes on HRQOL domains, for both treatment pathways [95]. Further insight into patients’ biopsychosocial health status and frailty is also necessary, both in daily CKD care to inform decision-making and in research to enable more accurate comparisons and predictions of outcomes between patient groups on different treatment pathways. To achieve this, an NGA is required to collect these data in clinical practice, whilst future research should focus on identifying the essential elements of patient characteristics and clinical tests that are minimally needed to come to a comprehensive, standardized, yet feasible NGA. Ideally, research should also aim to identify modifiable risk factors in individual patients, facilitating potential improvements in patients’ health outcomes.

Several prediction tools have been developed to assist patients and healthcare professionals in making well-informed choices between starting KRT or opting for CC. However, these tools typically focus only on survival outcomes, with most predicting mortality risk in dialysis and some requiring further external validation and refinement [96,97]. Additionally, it is necessary to establish practices for interpreting and discussing predicted risks with individual patients during decision-making [98]. Several international initiatives and multi-stakeholder projects have identified these issues as research priorities in geriatric nephrology [13,16,99,100]. Currently, an ongoing RCT in the UK is comparing HRQOL and survival outcomes for older patients with CKD on different treatment pathways [101]. Additionally, prospective cohort studies, such as the DIALOGICA and DOMESTICA studies in the Netherlands and the GOAL study in Australia, are underway to increase our understanding of outcomes in older patients on both treatment pathways and to identify key components of an NGA [73,79,102]. Other research initiatives are focusing on the development and implementation of patient decision aids, aiming to translate growing evidence on outcomes into daily clinical care and support SDM and ACP for individual patients [103,104].

While the shifts toward nephrogeriatric care, including the use of an NGA and the measurement of more patient-relevant outcomes, helps enhance our understanding of the patient’s perspective, further analysis is needed to determine whether these strategies and outcomes are truly person-centered and promote SDM [105,106]. For instance, the increasing use of PROMs to assess health outcomes may bring to light new topics that matter to patients. However, most PROMs still have a biomedical orientation [107]. Moreover, while an NGA is based on a biopsychosocial model of health rather than a purely biomedical one, it may still miss aspects of the patient’s perspective regarding what truly matters to them in living a meaningful life. For instance, it has been suggested that the concept of existentiality and life goals should be incorporated as a fifth domain within the NGA, explored alongside the patient.

In the context of complex chronic care, such as in older and frail patients with CKD, additional strategies may be needed to ask the right questions, focusing on what truly matters to these patients [108,109,110].

Collaborative care between nephrologists, geriatricians and palliative care experts could help shift the focus from disease and pathophysiology (“*What is the matter with you?*”) and the aim to fix that to the patient’s values and preferences (“*What matters to you?*”). This approach allows for determining the best course of action given the patient’s individual situation, especially when multiple comorbidities are involved. Adapted SDM models that prioritize exploring and establishing the goals of care first have been recommended for such patient populations [111,112]. The setting of CKD offers valuable opportunities for this decision-making process, as it often provides time and the chance to build long-term relationships between older patients and their MDTs, based on person-centered partnerships [108].

### 7.2. Integrating All Relevant Perspectives

Multidisciplinary collaboration is essential for integrating diverse expertise and delivering person-centered, biopsychosocial care. Equally important is involving patients and their representatives as active partners in care decisions to ensure alignment with their values and goals. Incorporating NGA during decision-making and fostering collaboration among specialists allows for a comprehensive understanding of patient needs. This patient-centered approach shifts the focus from disease management to improving outcomes and enhancing the HRQOL for older patients with CKD.

## 8. Conclusions

In conclusion, optimizing CKD care for older patients requires a shift toward person-centered, biopsychosocial care. Collaborative efforts, GA integration, and SDM ensure that care reflects the patient’s priorities and goals.

## Figures and Tables

**Table 1:** Overview of national guidelines, current practice in CKD care and patient resources in five European countries.

	National Guidelines			Current Practice in CKD Care		Patient Resources	
	CKD + SDM	CC	PC	MDT	GA	Online Information	Decision Support Tools
The Netherlands	+	+	+	+	NRR	+	+
Italy	*	*	+	+	NRR	+	-
United Kingdom	+	+	−	+	NRR	+	+
France	+	+	−	+	NRR	+	−
Belgium	*	*	−	+	NRR	+	−

+ = Available; − = Not available; * International guidelines such as those of the KDIGO, NICE, and ERBP are used; CKD = chronic kidney disease; SDM = shared decision-making; CC = conservative care; PC = palliative care; MDT = multidisciplinary care team including a nephrologist, specialized nurse, social worker, dietician; GA = geriatric assessment; NRR = not routinely recommended. References to guidelines and website links are available in Supplementary Table S1.

## Supplementary Materials

The following supporting information can be downloaded at: https://www.mdpi.com/article/10.3390/jcm14051749/s1. Figure S1. Dutch decision aid for patients (www.nvn.nl); Table S1: References to Guidelines and Website Links for Current CKD Care Practice and Patient Resources in Five European Countries.
