# Supplementary material for: Geriatric Assessment in Older Patients with Advanced Kidney Disease: A Key to Personalized Care and Shared Decision-Making—A Narrative Review
Source: J Clin Med. 2025 Mar 5;14(5):1749. doi: 10.3390/jcm14051749 (PMC11900943; doi:10.3390/jcm14051749)
Supplement: Supplementary file 1 [file jcm-14-01749-s001.zip › jcm-3468565-supplementary.pdf]

Supplementary Materials

CURRENT CKD CARE PRACTICE IN 5 EUROPEAN COUNTRIES

After outlining the theoretical model to optimise care for older patients with chronic kidney disease (CKD), we reviewed current practices, national guidelines, and patient resources across five European countries. We drew on our own clinical expertise, supplemented by outreach within our professional network and targeted internet searches. The key findings are summarized in the main review, while more detailed information, including guideline references and relevant website links, is provided in this supplemental material.

1. THE NETHERLANDS

In the Netherlands, the Dutch kidney foundation (Nierstichting Nederland) and the kidney patient association (Nierpatienten Vereniging Nederland) (1, 2) have collaborated to create an extensive collection of resources for kidney replacement therapy (KRT) and its implications on patients' daily life. These resources cover all aspects of kidney failure, treatment modalities, instructions on how to properly use medication and emphasize the importance of healthy living. Information is available both online and in print. Additionally peer educators are available to help fellow patients in the difficult choices they have to make regarding initiating dialysis or undergoing a kidney transplantation (KT).

Efforts have been made to empower patients more and encourage them to become active participants in their kidney care, alongside their caregivers and professional healthcare team. The Dutch patient federation has launched an campaign to enhance patient participation and improve shared decision-making (SDM) between patients and healthcare professionals (3). As part of this campaign, patients are encouraged to ask 'three essential questions' (Drie Goede Vragen) during each clinic visit:

Patients are told to ask '3 good questions' (Drie Goede Vragen) at each clinic visit.

1) What are my treatment options?
2) What are the potential benefits and disadvantages of these options?
3) What does this mean for my personal situation?

Several guidelines in the Netherlands address the treatment of older CKD patients approaching end-stage kidney disease (ESKD) and guide the decision-making process regarding the initiation of KRT. These guidelines consider the overall functioning of the older patient, geriatric assessment (GA) in ESKD, and the incorporation of palliative care.

1.1 Guideline for the functioning of older kidney patients (4)

Most patients with kidney failure (eGFR <20-30ml/min/1.73m^2), are referred to kidney failure clinics (pre-dialysis clinics) to assist them in the decision making process. The multidisciplinary care team (MDT), including nephrologists, specialized nurses, social workers and dieticians, collaborate closely with patients.

The choice of treatment (KT, peritoneal dialysis (PD), at home or in center haemodialysis (HD) or conservative care (CC)) depends mainly on patient preferences and medical contraindications. The MDT is helps guide patients in this decision-making process.

The decision to start KRT is influenced by several factors, including patient preferences, comorbidities and cognitive and physical status. Age is not a primary determinant; instead, frailty is given greater weight when assessing the treatment burden and potential benefits of KRT. Guidelines do not set strict age limits for starting KRT but recommend caution when considering KRT for patients over 80 years of age, given the limited reported benefits in this age group.

Healthcare professionals are trained in SDM, using the three-phase Elwyn model: choice talk, option talk and decision talk. This process provides patients with detailed information about KRT, using decision support aids for patients developed by the Dutch patient and healthcare organizations (***Supplemental Figure S1***). These aids are available both online and in print for patients less familiar with digital tools.

The starting point is that all treatment options are equally valid, and patients should select the dialysis modality that best suits their personal situation. It is also recommended that life partners and family members be included in the decision making process, as the impact of kidney failure and KRT extends to caregivers as well.

1.2 Guideline on geriatric assessment in ESKD (5)

It is recommended to assess older patients with kidney failure for frailty using validated instruments, such as the Lawton Instrumental Activities of Daily Living (IADL) scale or the Katz Activities of Daily Living (ADL) scale. Risks associated with frailty and physical, cognitive and social impairments should be considered in patients choosing optimal KRT or CC. High levels of frailty and poor physical and cognitive functioning are associated with higher complication rates, functional decline and reduced health-related quality of life

(HRQOL) after starting KRT. However, no randomized controlled trials (RCTs) or prediction models are available to guide the selection of patients who would benefit more from KRT compared to CC. Existing data only show associations between functional status and poor outcomes. Therefore, it is recommended to assess comorbidities using a tool such as the Charlson Comorbidity Index, which helps identify patients at higher risk of mortality. Patients with a high risk of death within the next 6 to 12 months should be considered for CC instead of starting dialysis or undergoing KT. Additionally, patients with advanced age and severe chronic malnutrition are unlikely to benefit from KRT.

A comprehensive geriatric assessment (CGA) is recommended as part of the pre-KRT evaluation for older patients. However, due to the labour-intensive nature of CGA, Dutch guidelines do not recommend its routine use in all CKD patients. It is advised, instead, to conduct a CGA when deciding whether to initiate dialysis or continue with CC. However, nephrologists are generally not trained in performing CGA.

All relevant factors should be discussed openly with patients, including the potential lack of survival benefits after starting KRT compared to CC. Studies indicate that patients who receive comprehensive education during the pre-dialysis phase are more likely to feel satisfied with their treatment decisions and experience lower levels of decision regret.

1.3 Guideline on palliative care in ESKD (6)

The World Health Organization (WHO) defines palliative care as an approach that aims to prevent and alleviate health-related suffering in adults, children and their families, facing problems associated with life-threatening illness. Palliative care is based on a comprehensive and person-centered approach, addressing physical, psychosocial, social and spiritual suffering (7).

Palliative care in nephrology focusses on four dimensions: physical, social, psychosocial and spiritual wellbeing. It is delivered by MDT, with patients at the center of care. The impact of CKD on family members and caregivers must also be considered, as living with a chronic disease can be a significant burden. Palliative care involves both treating the underlying illness and alleviating symptoms. As CKD is incurable and a lifelong diagnosis, one could state that all patients with CKD that are not eligible for KT, should receive palliative care.

As kidney failure progresses and age advances, treatment goals may shift over time. The number of patients choosing CC increases steadily. The main goals of CC are to slow the progression of kidney failure, manage the symptoms associated with kidney failure, and to optimize HRQOL. These goals can only be achieved through collaborative efforts of MDTs, in cooperation with general practitioners.

Guidelines recommend that all four aspects of palliative care be considered when treating patients with kidney failure. An individualized care plan should be developed, based on the patient's needs and preferences. The patient must remain at the center of this plan, with ongoing discussions about treatment goals and limitations, such as refraining from hospitalization or resuscitation. Care plans should be regularly reviewed and adjusted, at least annually or whenever there is a significant change in the patient's physical or cognitive condition. A useful tool to mark the beginning of the palliative phase is the 'surprise question': '*Would I be surprised if this patients dies within the next 6-12 months?'* If the answer is '*no*', the care plan should be revised. Nephrologists should regularly ask this question when assessing older patients, as frailty is strongly associated with increased mortality.

Family members and caregivers should be involved in the evaluation of care plans. Preparing for future events, such as the patient's preferred place of death, is an essential component of advance care planning (ACP), which should be discussed both with patients who are on dialysis and those who have opted for CC. If dialysis is discontinued, death typically occurs within one to three weeks, depending on residual kidney function, comorbidities, and physical status. This is an important topic to address with both patients and their caregivers at home.

To help professional healthcare teams and patients, brochures are available to guide patients through the palliative and end-of-life phases.
Palliative care is delivered by MDTs in various settings, including hospitals, care facilities and at home. Effective communication and transfer of information – whether in person and/or in writing - are therefore essential for providing optimal care for these patients.

Supplemental Figure S1. Dutch decision aid for patients (www.nvn.nl)

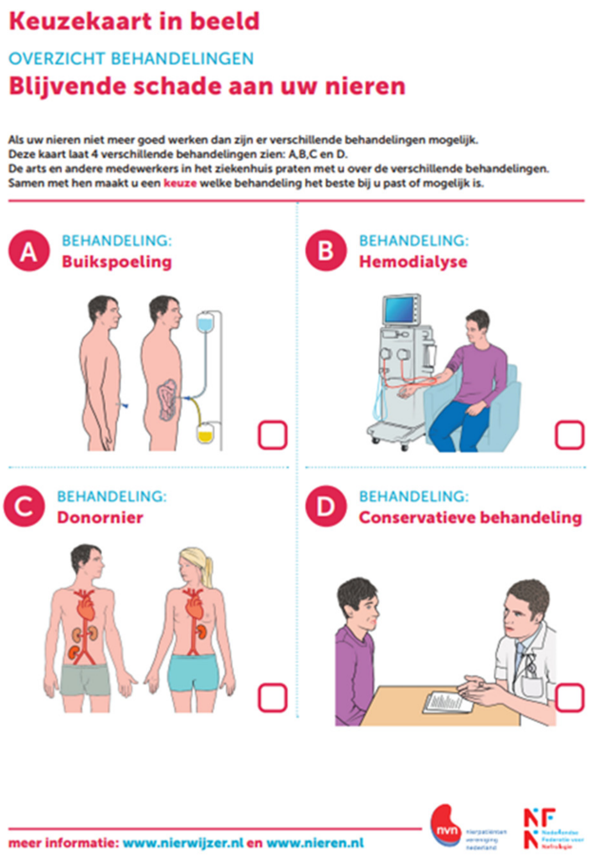

2. ITALY

We only found one national guideline (8). The primary focus of this guideline is the timely identification of patients requiring palliative care. Various tools or instruments can assist in this process, such as the Cohen's index (the surprise question) for incident dialysis patients or Couchoud's model for patients with renal failure. Additionally, patients with reduced life expectancy or a higher risk of complications should be identified. For this purpose, the Multidimensional Prognostic Index (MPI), a validated tool for older patients with chronic conditions is recommended. The MPI evaluates several aspects, including:

- Functional status (ADL and IADL)
- Cognitive status (SPMSQ: Short Portable Mental Status Questionnaire)
- Comorbidities (CIRS: Cumulative Illness Rating Scale)
- Nutritional status (MNS: Mini Nutritional Assessment)
- Decubitus risk (Exton-Smith Scale)
- Social environment
- Polypharmacy

Furthermore, illness-related symptoms and psychological factors should be considered when selecting patients for palliative care.

The next step involves a comprehensive, multidimensional assessment to determine the patient's needs, treatment goals, and preferred lifestyle while managing renal failure. This assessment should also encompass the needs of partners and family members. The result is a tailored care plan that ensures the delivery of the appropriate care, at the right time, and in the preferred setting.

Effective palliative care requires a multidisciplinary team trained and experienced in this field. Care plans should be reviewed and updated regularly to reflect any changes in the patient's condition, goals, or needs. Early identification of patients at high risk of complications or with a limited short-term prognosis is crucial for timely plan adjustments.

Ethical considerations and adherence to national laws and regulations are essential in providing palliative care. The guidelines include seven recommendations that align with these principles. Shared decision-making (SDM) is integral to the process, requiring regular, scheduled discussions with patients and their families. These discussions should cover the disease progression, expected outcomes, and care goals while allowing space for emotions and concerns to emerge.

Care plans should be discussed and documented in the patients' files in order to be accessed by all members of the renal care team.
Care plans must be documented in the patient's medical record to ensure all members of the MDT have access. Decisions regarding initiating or discontinuing dialysis should follow best clinical practices and may do no further harm to patients. If dialysis prolongs suffering or fails to improve symptoms, it should be withheld or discontinued, with decisions guided by the patient's wishes and the physician's clinical judgment. For patients unable to make decisions, family members or legal guardians, in collaboration with the MDT, should determine the best course of action. In cases of uncertainty regarding the benefits of dialysis, a trial period of up to four months may be considered. If no significant improvements are observed, continuing dialysis is not recommended.
Patients with kidney failure often spend considerable time in hospitals during their last months of life, with many dying in hospital despite expressing a preference to die at home. A well-structured palliative care plan can help patients live their remaining time in accordance with their wishes and support their families during the grieving process. End-of-life care should prioritize symptom management, effective pain relief, and psychological support for both patients and their loved ones.

3. UNITED KINGDOM

3.1 Guidelines on CKD management

There are several comprehensive guidelines in the UK for managing CKD. The National Institute for Health and Care Excellent (NICE) provides guidance through the following:

- Guideline on Chronic Kidney Disease Assessment and Management (NG203) (9): covers diagnosis, monitoring and management of CKD and its complications.
- Guideline on Renal Replacement Therapy and Conservative Management (NG107 (10): covers starting, stopping and switching treatments, including a focus on SDM.

Additionally, the UK Kidney Association offers a range of guidelines in managing different aspects of CKD such as anaemia, exercise and nutrition, and haemodialysis (11). It also provides an online resource, the 'eCKD Guide', for accessible, evidence-based information.

3.2 Guidelines on supportive care and nephrogeriatric liaison

There is less guidance specifically on supportive care in kidney disease, or nephrogeriatric involvement. The NICE guideline (NG203) on CKD Assessment and Management highlights the need to consider frailty during medicine optimisation and blood pressure management. Similarly, the guideline on 'Renal Replacement Therapy and Conservative Management' (NG107) alludes to the lack of evidence of harms or benefits in any specific group of people, and comments that decisions for dialysis or CC should be based on individual factors such as frailty, cognitive impairment, and multimorbidity. However, there is no specific direction for targeted NGA.

National policy and agenda is shifting towards the needs of an ageing, frailer UK population with CKD. For example, an NHS initiative called GIRFT (Getting it Right First Time) (12) aims at standardising care and reducing variability across the UK, using a data driven approach, and makes recommendations for improving care and efficiency. A report in Renal Medicine in (13) 2021 highlighted the need to prioritise shared decision-making in the Advanced Kidney Care setting, ensuring all patients have access to a multiprofessional conservative care pathway if needed. As a result of this report, local renal networks have been established across the UK, many with dedicated supportive and conservative care workstreams, and with freely available resources in ACP and supportive care provision. (e.g. https://londonkidneynetwork.nhs.uk/supportive-care-workstream, https://wkn.nhs.wales/patients-carers/explore-my-treatment-options/supportive-care).

4. FRANCE

4.1. National guideline on CKD management (14)

France's national guideline for CKD offers comprehensive recommendations for managing adult patients. Key components include patient education, psycho-social evaluation, recommendation for formal geriatric assessment when a geriatric syndrome (e.g. neurocognitive impairment, malnutrition or functional decline) is suspected. Pharmacological strategies are outlined to slow disease progression and manage complications including anemia, hypertension, and comorbid conditions such as diabetes. Guidance is also provided for preparing patients for KRT.

A dedicated section focuses on managing older patients with CKD, particularly those over 80 or with vulnerabilities like dementia or functional decline. Due to limited validated clinical data, these recommendations are largely based on expert consensus.

CKD care coordination is led by the nephrologist working alongside a dietician, psychologist, nurses, social worker, and the general practitioner (GP). Discussions regarding KRT typically begin for patients with a creatinine clearance below 20 mL/min/1.73 m2, with specialized nurses, psychologists, and the GP progressively involved. For patients with frailty or geriatric syndromes, geriatricians may contribute to decision-making about KRT or CC, although their routine involvement remains limited.

4.2 National guideline for CC in ESKD (15)

Another guideline addresses CC for ESKD, offering guidance on communication strategies, discussion timing, and engagement of multidisciplinary teams, including home care nurses, palliative care specialists, and geriatricians. Symptom management and CGA are emphasized to tailor care to individual patient needs. Nephrologists maintain a central role in CC planning.

The Société Francophone de Néphrologie, Dialyse et Transplantation (SFNDT) further supports shared decision-making regarding dialysis and end-of-life care, promoting personalized therapy adjustments (16). No specific palliative care guideline for patients with ESKD is available.

5. BELGIUM

In Belgium, there are no national guidelines specifically addressing CKD treatment or palliative care for ESKD. Nephrologists generally rely on international KDIGO guidelines (17) along with Dutch and French resources, as outlined in earlier sections. For older patients with CKD, the European Renal Best Practice (ERBP) guideline (18) is commonly used, which advocates a personalized, patient-centered approach to care. This includes careful consideration of overall health, comorbidities, functional status, and patient preferences when determining treatment options. The guideline emphasizes tailoring dialysis or CC strategies based on life expectancy, QOL, and the balance of treatment risks and benefits. It also highlights the importance of SDM and effective communication between healthcare providers and patients. In Flanders, patient education initiatives are evolving, including the upcoming website info.zorgvoorjenieren.be, which will provide resources on CKD (19).

Decisions regarding the initiation of KRT are typically made in collaboration between the nephrologist and the patient, with attention to factors such as cognitive and functional status and overall QOL. However, screening tools for frailty, survival prediction models or a formal CGA are not routinely used in this process. Geriatricians are generally not involved in decision-making for KRT initiation. During the pre-dialysis phase, a multidisciplinary team—comprising nephrologists, dieticians, social workers, and dialysis nurses—evaluates KRT and CC options. Despite this collaborative approach, the inclusion of geriatricians or specialized geriatric nurses remains rare.

Supplemental Table S1

References to Guidelines and Website Links for Current CKD Care Practice and Patient Resources in Five European Countries

	National guidelines			Current practice in CKD care		Patient resources	
	CKD + SDM	CC	PC	MDT	GA	Online information	Decission support
**The Netherlands**	a+b+c	d	e	+	NRR	f+g	g+h
**Italy**	*	*	i	+	NRR	j	-
**United Kingdom**	k	l	-	+	NRR	m+n	o+p
**France**	q+r	s	-	+	NRR	t	-
**Belgium**	*	*	-	+	NRR	u	-

+ = Available; - = Not available; * International guidelines such as KDIGO, NICE, ERBP are used; CDK = chronic kidney disease; SDM = shared decision-making; CC = conservative care; PC = palliative care; MDT = multidisciplinary team including a nephrologist, specialized nurse, social worker, dietician; GA = geriatric assessment; NRR = not routinely recommended

a https://richtlijnendatabase.nl/richtlijn/chronische_nierschade_cns

b https://richtlijnendatabase.nl/richtlijn/nierfunctievervangende_behandeling

c https://richtlijnendatabase.nl/richtlijn/zorg_bij_eindstadium_nierfalen/geriatrisch_assessment_bij_eindstadium_nierfalen.html

d https://richtlijnendatabase.nl/richtlijn/zorg_bij_eindstadium_nierfalen/conservatief_beleid_bij_eindstadium_nierfalen.html

e https://richtlijnendatabase.nl/richtlijn/nierfalen/organisatie_van_zorg.html

f https://www.nieren.nl

g https://www.nvn.nl

h https://www.patientenfederatie.nl/wachtkamerfilmpjes/stel-de-3-goede-vragen

i https://www.sicp.it/wp-content/uploads/2018/12/26_Documento_SICP-SIN_edited_v2.pdf

j https://www.fondazioneitalianadelrene.org/

k https://www.nice.org.uk/guidance/ng203

l https://www.nice.org.uk/guidance/ng107

m https://www.nhs.uk/conditions/kidney-disease/

n https://kidneycareuk.org/

o Prichard, Alison & Thomas, Nerys. (2013). The option grid: a shared decision-making tool for renal patients. Journal of Renal Nursing. 5. 6-11. 10.12968/jorn.2013.5.1.6.

p Winterbottom AE, Mooney A, Russon L, Hipkiss V, Ziegler L, Williams R, Finderup J, Bekker HL. Kidney disease pathways, options and decisions: an environmental scan of international patient decision aids. Nephrol Dial Transplant. 2020 Dec 4;35(12):2072-2082.

q https://www.has-sante.fr/upload/docs/application/pdf/2021-09/guide__mrc.pdf

r Groupe de travail de la Société francophone de néphrologie, dialyse et transplantation (SFNDT). Adaptation raisonnée des thérapeutiques (ART en néphrologie) - Guide pratique LAT-MRC1 [Withholding or withdrawing life support in nephrology: a perspective in the French-speaking background]. Nephrol Ther. 2024 Jun 26;20(3):1-34.

s Groupe de travail de la SFNDT. Traitement conservateur de la maladie rénale chronique stade 5 : guide pratique [Comprehensive conservative care of stage 5-CKD: A practical guide]. Nephrol Ther. 2022 Jun;18(3):155-171.

t https://www.francerein.org/

u https://info.zorgvoorjenieren.be

References

1. Nierstichting en nierpatiënten vereniging Nederland. Kennis, steun en inspiratie over leven met een nierziekte n.d. [Available from: https://www.nieren.nl.
2. Nierpatiënten vereniging Nederland. Patiënteninformatie en -ondersteuning n.d. [Available from: https://www.nvn.nl.
3. Patiëntenfederatie Nederland. Stel de 3 goede vragen n.d. [Available from: https://www.patientenfederatie.nl/wachtkamerfilmpjes/stel-de-3-goede-vragen.
4. Federatie Medisch Specialisten. Nierfunctievervangende behandeling 2016 [Available from: https://richtlijnendatabase.nl/richtlijn/nierfunctievervangende_behandeling/nierfunctievervangende_behandeling_-_startpagina.html.
5. Federatie Medisch Specialisten. Zorg bij eindstadium nierfalen 2020 [Available from: https://richtlijnendatabase.nl/richtlijn/zorg_bij_eindstadium_nierfalen/startpagina_-_zorg_bij_eindstadium_nierfalen.html.
6. Federatie Medisch Specialisten. Palliatieve zorg bij eindstadium nierfalen 2017 [Available from: https://richtlijnendatabase.nl/richtlijn/nierfalen/inleiding.html.
7. World Health Organization. Palliative care 2023 [Available from: https://www.who.int/europe/news-room/fact-sheets/items/palliative-care.
8. SICP E SIN. Le cure palliative nelle persone con mallatia renale cronica avanzata 2018 [Available from: https://www.sicp.it/wp-content/uploads/2018/12/26_Documento_SICP-SIN_edited_v2.pdf.
9. National Institute for Health and Care Excellence. Chronic kidney disease: assessment and management 2021 [Available from: https://www.nice.org.uk/guidance/ng203.
10. National Institute for Health and Care Excellence. Nice guideline [107] Renal replacement therapy and conservative management 2018 [Available from: https://www.nice.org.uk/guidance/ng107.
11. UK Kidney Association. Management of patients with CKD 2021-2025 [Available from: https://www/ukkidney.org/health-professionals/information-resources/uk-ecdk-guide/management-patients-ckd.
12. NHS England. GIRFT guide supports better care for people with kidney cancer 2023 [Available from: https://gettingitrightfirsttime.co.uk/girft-guide-support-better-care-for-people-with-kidney-cancer/.
13. NHS England. Renal Medicine: GIRFT programme National Specialty Report 2021 [Available from: https://gettingitrightfirsttime.co.uk/wp-content/uploads/2021/09/Renal-Medicine-Sept21k.pdf.
14. Haute Autorité De Santé. Guide du parcours de soins - Maladie rénale chronique de l'adulte (MRC) 2023 [Available from: https://www.has-sante.fr/upload/docs/application/pdf/2021-09/guide_mrc.pdf.
15. Groupe de travail de la SFNDT. Traitement conservateur de la maladie rénale chronique stade 5 : guide pratique [Comprehensive conservative care of stage 5-CKD: A practical guide]. NEPHROLOGIE ET THERAPEUTIQUE. 2022 Jun;18(3):155-71.
16. Société Gdtdl, francophone de néphrologie dialyse et transplantation (SFNDT). Adaptation raisonnée des thérapeutiques (ART en néphrologie) Guide pratique LAT-MRC 2024 [Available from: https://www.sfndt.org/sites/www.sfndt.org/files/medias/documents/N%26T%202024%20Guide%20ART%20en%20N%C3%A9phrologie.pdf.
17. Kidney Disease: Improving Global Outcomes (KDIGO) CKD Work Group. KDIGO 2024 Clinical Practice Guideline for the Evaluation and Management of Chronic Kidney Disease. KIDNEY INTERNATIONAL. 2024;105:S117-S314.
18. Farrington K, Covic A, Aucella F, Clyne N, de Vos L, Findlay A, et al. Clinical Practice Guideline on management of older patients with chronic kidney disease stage 3b or higher (eGFR <45 mL/min/1.73 m2). NEPHROLOGY DIALYSIS TRANSPLANTATION. 2016;31:1-66.
19. Nederlandstalige Belgische Vereniging voor Nefrologie. Ontdek meer over niergezondheid n.d. [Available from: https://www.info.zorgvoorjenieren.be.
